# Ontario Veterinary College

**Published:** 1899-05

**Authors:** 


### ONTARIO VETERINARY COLLEGE.

The closing exercises of the Ontario Veterinary College for the season of 1898–1899 were held in the college, Temperance Street, Toronto. Prof. A. Smith, F.R.C.V.S., occupied the chair, and with him on the platform were Mayor Shaw, Mr. J. J. Withrow, Mr. C. Elliott, V.S., of St. Catharine's; Dr. Duncan, Dr. Chambers, Dr. Amyot, and other members of the staff of the college. There was a full attendance of the students, anxious to know the results of the recent examination, their presence being manifested by frequent bursts of applause as the names of the successful students were announced. Dr. J. T. Duncan read the list of graduates and prize-winners. His Worship the Mayor, on rising to address the students, was received with cheers. He said he claimed also to be a student, but it was of municipal politics, probably almost as difficult a branch of knowledge as that of the sciences. He was delighted to see such a number of students hailing from the adjoining republic, and he felt sure that they would carry home with them pleasant memories of the time they had spent in Toronto. He hoped and believed that the cordial relations that existed between Canada and the United States would always continue to the mutual benefit of both. Mr. C. Elliott, V.S., one of the Board of Examiners, in congratulating the graduating class, said that it compared quite favorably with the classes of previous years. He also urged on the graduates the necessity of continuing their studies.

Mr. Cowan, V.S., also of the Board of Examiners, gave a short and useful address, containing much good advice to those about to enter on their chosen life-work.

Mr. Withrow said it was by no means the first time that he had addressed the graduating class of the Ontario Veterinary College, and he had often wondered where the graduates all went to. He was much impressed with the remark of Mr. Cowan, the previous speaker, that the student who passed a few years ago was not as well fitted for his life's work as those who have passed to-day unless he has studied and kept up with the times; so he strongly advised constant application to acquire the most recent and advanced knowledge.

Mr. Sewell, president of the graduating class, presented Prof. Andrew Smith with a beautiful picture of the graduating class as an acknowledgment of the kindness and attention they had received during their attendance at the college. He also made a graceful allusion to the recent death of an old and respected member of the Board of Examiners, Mr. Wilson, V.S., of London.

Prof. Smith thanked Mr. Sewell, as representing the graduates, for his words of appreciation. He also spoke of the pleasant associations connected with this and other classes, which the picture would call to remembrance. He referred with much feeling to the death of his old friend, Mr. Wilson, to whom Mr. Sewell referred, and in conclusion he wished them every success in the future.

The following is the full list of graduates:

Victor J. Andre, St. Genevieve, Mo.; H. W. Carley-Baker, London, Eng.; John H. Black, Toronto; J. Alex. S. Berryman, St. John, N. B.; John W. Corrigan, Owego, N. Y.; C. J. Donnelly, Orilla; Fred. B. Davidson, Glencoe; Wilfred J. R. Fowler, Seaforth; Charles T. Frerg, Warwick, R. I.; John J. Giblin, Blackstone, Mass.; Wesley M. Goff, Lewiston, Maine; William J. Hennessey, Worcester, Mass.; Arthur Hobbs, Carlisle, Eng.; Duncan A. Irvine, Dalkeith; George Jerome, Rapid River, Mich.; Fred. Clee Jones, Birmingham, Eng.; Arthur E. Joslin, Adrian, Mich.; Lewis B. Judson, Bethel, Conn.; W. Luther Jones, Edgefield, South Carolina; Harvey F. Kaufman, Hegins, Pa.; Joseph King, Brice, Ohio; Thomas J. Kirwan, Auburn, N. Y.; William A. Kuhns, Boston, Mass.; Charlie A. Locke, Trilly, N. Y.; John McDougall, Ralphton, Man.; James P. McVicar, Petrolea; John M. Mitchell, Morristown, N. J.; W. H. Murphy, Jr., Brighton, Mass.; Charles L. Manning, Grand Ledge, Mich.; Patrick H. Morin, Port Colborne; F. J. Neff, Long Glade, Virginia; Jacob F. Olweiler, Elizabethtown, Pa.; Herbert J. Pugsley, Central Cambridge, N. B.; John W. Purdy, Oliver, Ont.; Howard O. Ramsey, Merle, Cal.; Ulysses S. Richards, Lowell, Mass.; Albert J. Roll, Natrona, Pa.; William L. Rundle, Chapman Quarries, Pa.; John Russell, Saginaw, Mich.; John A. Scott, Grand Rapids, Mich.; J. Lee Shorey, Hoosick Falls, N. Y.; John E. Sommer, Buffalo, N. Y.; W. A. Sproule, Boissevain, Man.; J. M. Sewell, Bunker Hill, Ill.; Edward M. Saigeon, Lapeer, Mich.; William Benjamin Wentzell, Amherst, Mass.; James M. Young, Petrolea.

